# Gene expression changes within Müller glial cells in retinitis pigmentosa

**Published:** 2012-05-09

**Authors:** Karin Roesch, Michael B. Stadler, Constance L. Cepko

**Affiliations:** 1 Department of Genetics, Harvard Medical School, and Howard Hughes Medical Institute, Boston MA; 2 Friedrich Miescher Institute for Biomedical Research, Basel, Switzerland

## Abstract

**Purpose:**

Retinitis pigmentosa (RP) is a progressive retinal degeneration in which the retina loses nearly all of its photoreceptor cells and undergoes major structural changes. Little is known regarding the role the resident glia, the Müller glia, play in the progression of the disease. In this article, we define gene expression changes in Müller glial cells (MGCs) from two different mouse models of RP, the retinal degeneration 1 (rd1) and rhodopsin knockout (Rhod-ko) models. The RNA repertoire of single MGCs was comprehensively profiled, and a comparison was made between MGCs from wild-type (WT) and mutant retinas. Two time points were chosen for analysis, one at the peak of rod photoreceptor death and one during the period of cone photoreceptor death.

**Methods:**

Retinas were dissociated, and single MGCs were chosen under a dissecting microscope using a micropipette. Single cell cDNAs were generated and genome-wide profiles were obtained by hybridization to Affymetrix arrays. A comparison was made among all samples to discover the changes in gene expression during the periods of rod and cone photoreceptor death.

**Results:**

MGCs respond to retinal degeneration by undergoing gliosis, a process marked by the upregulation of glial fibrillary acidic protein (*Gfap*). Many additional transcripts were found to change. These can be placed into functional clusters, such as retinal remodeling, stress response, and immune-related response.

**Conclusions:**

A high degree of heterogeneity among the individual cells was observed, possibly due to their different spatial proximities to dying cells and/or inherent heterogeneity among MGCs.

## Introduction

Many degenerative diseases of the retina involve the loss of photoreceptor cells. These diseases most often are due to mutations that directly affect the photoreceptor cells (RetNet). Other cell types, such as neurons that are postsynaptic to photoreceptor cells, as well as glial cells, are indirectly affected by these changes within photoreceptor cells. Müller glial cells (MGCs) are the predominant type of glial cell in the vertebrate retina. They are likely sensitive to changes in the status of photoreceptor cells, as MGC processes ensheathe the photoreceptors, and MGCs offer various types of support to the photoreceptor cells (reviewed in [1]). Müller glial cell bodies localize to the middle of the inner nuclear layer. In addition to processes that ensheathe the photoreceptors, MGC radial cell processes span the entire retina. In contrast to glial cells in other regions of the central nervous system, where the cells greatly outnumber neurons, MGCs account for only 2% to 5% of the total retinal cell population [2,3]. In the healthy retina, they contribute to various essential functions, including structural support to neurons, neurotransmitter recycling, regulation of ion homeostasis, glia-neuron communication, glutamate metabolism, maintenance of the blood–retinal barrier, and energy provision [1,4,5]. They also have been reported to function as light pipes that transmit light across the retina to the photoreceptors [6]. MGCs are dynamic cells that express many receptors that enable the cells to respond to their environment [4]. They can react to various molecules such as neurotransmitters, growth factors, and cytokines. MGCs can also modify neuronal activity by secreting various growth and neurotrophic factors [7-12].

MGCs respond to insults to the retina through a process referred to as gliosis [1,13]. The function of gliosis is poorly understood. Even fundamental aspects, such as whether gliosis is protective or destructive, are not understood [1,14]. No studies have been performed to systematically characterize the gene expression response of MGCs during the progress of retinitis pigmentosa (RP), a common inherited disorder of the retina (Retnet). The most well known aspect of the glial response is that MGCs upregulate the intermediate filament protein, the glial fibrillary acidic protein (GFAP), a protein considered a marker for reactive gliosis not just in the retina but across the entire central nervous system (CNS) [13,15]. GFAP expression is increased in response to a wide range of insults. In other areas of the CNS, gliosis has been suggested to have numerous beneficial functions, such as providing a physical barrier that protects tissue from further damage [16-20]. However, studies also have suggested detrimental effects, such as inhibition of axon regeneration [20-22]. In the retina, the functional consequences of gliosis are unclear.

Retinitis pigmentosa (RP) is an inherited disorder characterized by an initial phase of rod photoreceptor dysfunction and death, followed by cone death. RP has an incidence of about 1 in 3,500–4,000 people [23]. Mutations have been identified in more than 40 genes, with many of these genes expressed only in rods. In fact, the locus with the highest number of disease alleles for the autosomal dominant form of RP is the rhodopsin locus. The current study used two animal models of RP, with mutations in two different rod-specific genes. One mutation is in the rhodopsin locus, where no protein is made (Rhod-ko) [24], and the other is in the *Pdeb* (phosphodiesterase beta) locus where a non-functional protein is made (rd1) [25]. The disease progression in the retina in the two models differs in the kinetics of degeneration. However, both have the same outcome, loss of photoreceptors resulting in a total collapse of the outer nuclear layer [24-26]. In a patient, these two phases of photoreceptor cell death correspond to an initial loss of night vision, followed by tunnel vision. Eventually, an individual with RP loses all vision.

To create a data set that can be further explored regarding the function of MGCs during gliosis, we sought to create a comprehensive catalog of changes in gene expression in MGCs during RP. As MGCs are only a small percentage of all cells in the retina [3], we chose to profile several single MGCs from wild-type (WT) and diseased retinas. The transcription profiles of single MGCs at two different stages of disease and in the two models of RP mentioned above were established using Affymetrix microarrays. The data from each cell were compared to profiles from WT controls, as well as each other’s profiles. We used the five previously published MGCs characterized by our laboratory as the adult WT controls [27]. We also profiled three additional WT glial cells picked from a WT strain congenic with the strain carrying the rd1 mutation. The results provide a survey of molecules with increased or decreased expression in MGCs during photoreceptor cell death. A substantial number of genes were found to be coregulated with the gliosis marker *Gfap*. We discuss how these changes might alter MGC activity and how these changes might affect the surrounding neurons. More detailed knowledge of the role of MGC gliosis during photoreceptor cell loss could potentially lead to novel concepts and ideas for therapeutic strategies for RP.

## Methods

### Animals

WT mice (C57/BL6) and rd1 (FVB) mice were purchased from Charles River Laboratories (Wilmington, MA). The rd1 mice carry a mutation in the β-subunit of cGMP phosphodiesterase (*PDE6B*). Congenic FVB mice were purchased from Jackson Laboratories (stock number 4828l, FVB.129P2-Pde6b^+^Tyr^c-ch^/AntJ; Bar Harbor, ME). The Rhodopsin knockout (Rhod-ko) mice are deleted in the rod-specific rhodopsin gene and were a gift from J. Lem (Tufts University, Boston, MA) [24].

### Isolation of single Müller glial cells and cDNA amplification

Isolation of single MGCs and cDNA amplification were performed as described in [27-31]. Retinas were dissected from several different litters of Rhod-ko and rd1 (FVB) animals at 8 and 25 weeks, and at 13 days postnatal (P13) and 5 weeks of age, respectively, as well as at P13 from the control congenic FVB mice. The retinas were dissociated with papain (Worthington Biochemical, Freehold, NJ) with gentle trituration. Dissociated cells were viewed under a dissecting microscope, and MGCs were selected as single cells based upon their distinct morphology. cDNAs were generated with reverse transcription using Superscript II (Invitrogen, La Jolla, CA) and a modified Oligo dT primer: TAT AGA ATT CGC GGC CGC TCG CGA TTT TTT TTT TTT TTT TTT TTT TTT (Oligos Etc., Wilsonville, OR), followed by poly(A) tailing using TdT (Roche, Nutley, NJ), and polymerase chain reaction (PCR) amplification using LA Taq polymerase (Takara Mirus Bio, Shiga, Japan) [31].

### Affymetrix array hybridization

Detailed procedures have been previously described in Trimarchi et al. [31] and Roesch et al. [27]. Briefly, samples were labeled with 25 uM biotin N6 ddATP and hybridized to mouse genome 430 2.0 Genechip oligonucleotide arrays (Affymetrix, Santa Clara, CA). The slides were scanned using a GeneChip Affymetrix Scanner 7G. Signals from microarrays were further processed by using Affymetrix MicroArray Suite Software (MAS 5.0). The resulting data were exported as tab delimited text files to Microsoft Excel (Microsoft Corporation, Redmond, WA; Microsoft Excel 2008 for mac, version 12.2.5). Appendix 1 lists the signal levels for all cells profiled in this study and the previously published WT MGCs [27] used for comparison. The raw and processed data files can be accessed through NCBI Gene Expression Omnibus (GEO) Series accession number GSE35386.

### Data analysis

A detailed description of Affymetrix data analysis has been previously published in Trimarchi et al. [31] and Roesch et al. [27]. Multiple probe sets corresponding to a single gene were combined by selecting whatever probe set gave the highest value in a particular cell, and median expression levels were calculated. From the distribution of median expression levels (x-Axis is logarithmic), an initial peak of low expressed genes (probably off) can be observed, followed by a tail on the right side (probably on; see “median expression distribution” in Appendix 2). Already the lowest median expression threshold of 500 separated this tail from the initial peak, and higher stringencies of 1,000 and 2,000 worked even better. From the initial approximate 20,000 genes, this filtering on the median expression retained about 6%–12% of the genes: 3,519 genes, 2,368 genes, and 1,508 genes for thresholds of 500, 1,000, and 2,000, respectively. To analyze the coregulated genes, a median expression threshold (i.e., at least half of the genes will be expressed at a higher level than the threshold) for an upregulated gene to be included in the analysis was set to 2,000, and any other gene that did not fulfill this criteria was filtered out. For downregulated genes, a median expression threshold of 2,000 had to be achieved by at least three out of five cells in the WT samples. The resulting scaled values were equally distributed into five bins. Probe set pairs were then analyzed for association using a contingency table, and a p value for significance was calculated using Fisher’s exact test. The data are represented in table format or visualized in Java TreeView (version 1.1.5r2) [32]. Tables contain the resulting p values of coregulation of any gene (row) with the gene of interest(s).

## Results and Discussion

### Gene expression changes during gliosis defined with microarray analysis

MGCs were isolated from dissociated retinas at several time points. As the morphology of single MGCs following dissociation is characteristic, this criterion was used for their selection [27]. The signals from the microarrays confirmed that these cells were indeed MGCs (e.g., see the signal for vimentin [*Vim*]). Single cells were collected at the peak of the rod and cone cell death phases. Death kinetics in both models in our colony have been previously established and described in Punzo et al. [26]. In the rd1 model, the peaks of the two cell death waves are at P12 and week 5. In the Rhod-ko model, the corresponding time points are 8 weeks and 25 weeks. To examine whether a known marker of gliosis, *Gfap*, was upregulated in the MGCs from the mutants, the RNA levels for this gene were examined. Robust expression of *Gfap* was found in most cells (four of five) from the Rhod-ko and rd1 model at the early time point. At the late time point in rd1, five of five cells expressed *Gfap*, and only one of five of Rhod-ko cells also had significant levels at the later time point. No RNA was detected in the WT cells at the adult stage (zero of five) or at P13 (zero of three) in the congenic rd1 animals.

**Figure 1:**
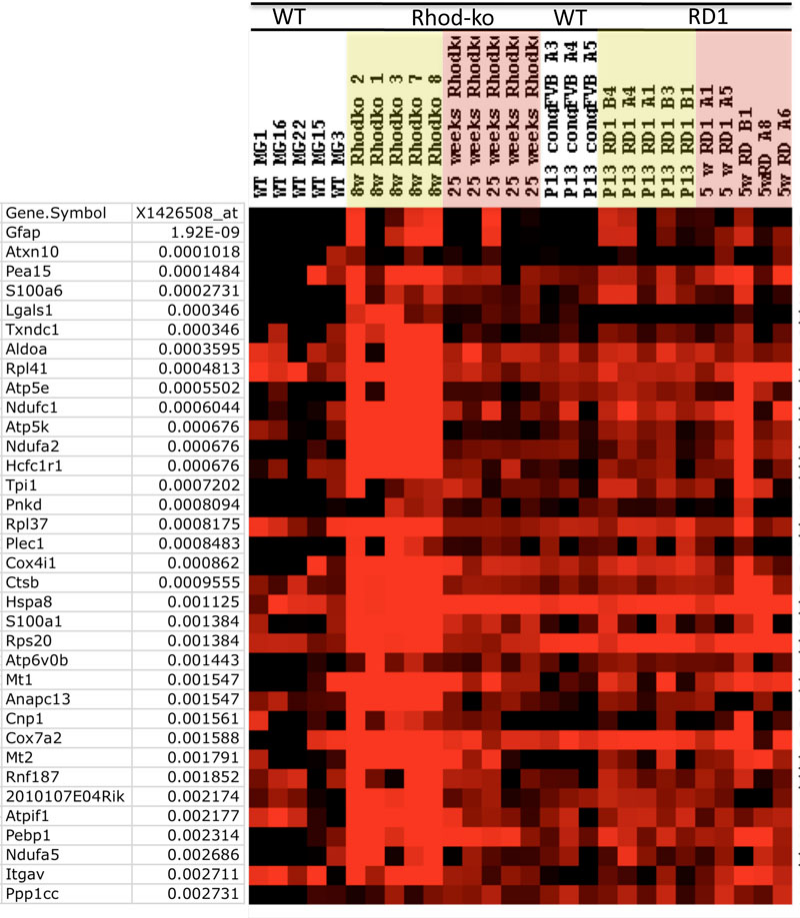
Single cell transcriptional profiles of genes highly associated with *Gfap*. Single MGCs were picked from dissociated retinas from two strains of degenerating retinas and from WT retinas. Single cell RNAs were reverse transcribed, amplified with PCR, and applied to Affymetrix microarrays. Columns represent the signal from single cell samples, rows represent genes. A heatmap was generated in TreeView such that bright red represents >10,000, and black <1,000, varying shades of red the values in between. A Fisher’s exact test was run to find genes that were coregulated with *Gfap* (Affymetrix identifier X1426508_at). All the top hits for genes coregulated with *Gfap* and their corresponding p values are shown. MGC samples: WT=wild-type, Rhod-ko=Rhodopsin knockout model, rd1=phosphodiesterase beta 1 rd1 mutant on the FVB strain background, and cong FVB is a WT control for rd1 on a congenic FVB background. Time points chosen are >21 days for WT, and 8 weeks and 25 weeks for the peaks of the two cell death waves in the Rhod-ko model. The corresponding time points in the rd1 are postnatal day P13 and 5 weeks. MGCs from congenic FVB at P13 were profiled as WT samples that are better age matched to the early cell death wave in the rd1 model.

To identify other genes induced during gliosis, we ran an analysis to look for genes coregulated with Gfap. The microarray signals for each gene across all cells were compared to the signals for Gfap in each cell. The probability that the observed pattern of expression for Gfap and each gene was by chance was computed using Fisher’s exact test. In Appendix 3 “p-vals distribution,” the number of genes coregulated with Gfap are plotted against their p values (Appendix 4, Appendix 5, Appendix 6). The expression levels for a selected subset of genes with significant p values are represented in detail as a heatmap in Figure 1. Clearly noticeable is a cluster of upregulated genes at the peak of rod cell loss in the Rhod-ko. Similar clusters, though at lower expression levels and more variegated, can be observed across the MGCs taken from the degenerating rd1 retinas at either time point. In the sections below, we discuss and speculate about the potential roles of the genes that exhibit changes in expression levels, as well as the difficulties encountered in our approach.

### Challenges in profiling single Müller glial cells

There are two advantages to using single MGCs for this analysis. As mentioned above, the percentage of MGCs in the WT retina is only 2%–5% [3]. Total tissue RNA preparations thus will not reveal genes that are at modest or low levels of expression within MGCs. Second, our previous single cell profiling of many types of retinal cells was undertaken with single retinal cells as we wished to examine cell types for their heterogeneity. As WT MGCs were included within this set, it was then a control set for comparing MGCs from diseased retinas. Examining the heterogeneity of response of MGCs to disease was also of interest. Previous studies, which characterized the entry of MGCs into the cell cycle following acute insults, showed that only a subset of MGCs responded by entering the cell cycle [33-39]. Single cell profiles might thus contribute to an understanding of this type of heterogeneity in the cellular response.

Only a handful of highly significant results were obtained (in the order of what is expected by chance given the p value cutoff). That does not necessarily mean that the results obtained are purely random—but that the type of data analyzed is rather noisy, thus limiting the statistical power. The obtained ranking of genes will still tend to put the biologically important ones toward the top of the list. In this case, there could be many false positives among the potentially true positives. However, there is also a difficulty inherent in using single cell profiling to unmask heterogeneity. If heterogeneity is significant, it presents a burden in statistical analysis. This problem is difficult to address because in the case of single cell expression data, replicates are not available. The low number of significant results in this data set might also depend on the additional filters applied (e.g., median expression level). This makes the analysis more stringent and goes beyond what the p value expresses. Nevertheless, it is a fair assumption that the data are relatively noisy and the statistical power is limited.

MGCs have been suggested to form functional columns with neurons as well as with each other [40-42]. In rodents, up to approximately 30 rods can be associated with one MGC [43]. Interestingly, cones do not belong to these columnar units, and are considered “extra-columnar” [43]. Within a column, MGCs are assumed to communicate with each other in coordinated ways via gap junctions [44]. MGCs can be recruited to or can leave the column, but neighboring columns do not necessarily have to provide the same function [45]. In keeping with this idea, we found significant heterogeneity among WT MGCs in our previous study [27]. Heterogeneity in disease response might then be expected due to this inherent heterogeneity among the WT cells. Additional variability likely would stem from the different ages and time points at which cells were collected in different mutant animals as well as from differences in the location of individual cells. Cell death in RP spreads from the center to the periphery of the retina, and therefore, cells originating from the center versus the periphery might differ. In the teleost retina, spatial diversity and heterogeneity have been previously suggested based on variable levels of proliferation among MGCs in correlation with the most severely damaged photoreceptors [46]. However, even in one location, not all MGCs enter into the cell cycle following an insult, suggesting heterogeneity in response even in one location [39,47,48].

Validation of the microarray results using in situ hybridization (ISH) for genes that change in MGCs during disease was undertaken. However, the results were difficult to interpret. Glial transcripts are dispersed throughout the cell body, resulting in ISH signals that are diffuse. Additionally, slow degeneration due to photoreceptor cell loss, particularly as occurs in the Rhod-ko retina, likely does not give striking changes in MGCs. Finally, staining observed at the inner and outer limiting membranes, structures defined by MGC processes, was difficult to assess, as non-specific staining was sometimes observed in these locations. All of these features made quantitative comparison among MGCs using ISH difficult. However, in our previous studies of many types of single retinal cells, we performed ISH using several hundred probes to examine gene expression patterns suggested by the microarrays [31,49]. In each case, the microarray data were shown to accurately represent the expression pattern. These data suggest that the changes observed among the microarrays in the current study likely reflect changes in gene expression during gliosis among some of the MGCs.

### Gene expression changes during gliosis: Possible functional consequences

The types of processes that one might predict would occur during photoreceptor death include removal of cell debris, restoration of homeostasis, arrest of secondary degeneration, sealing of the lesion site, and tissue reorganization. MGC gliosis likely contributes to some of these aspects. The gene expression changes seen here do in fact support these roles for MGCs during photoreceptor degeneration. The results discussed below focus on these features.

### Remodeling

**Figure 2:**
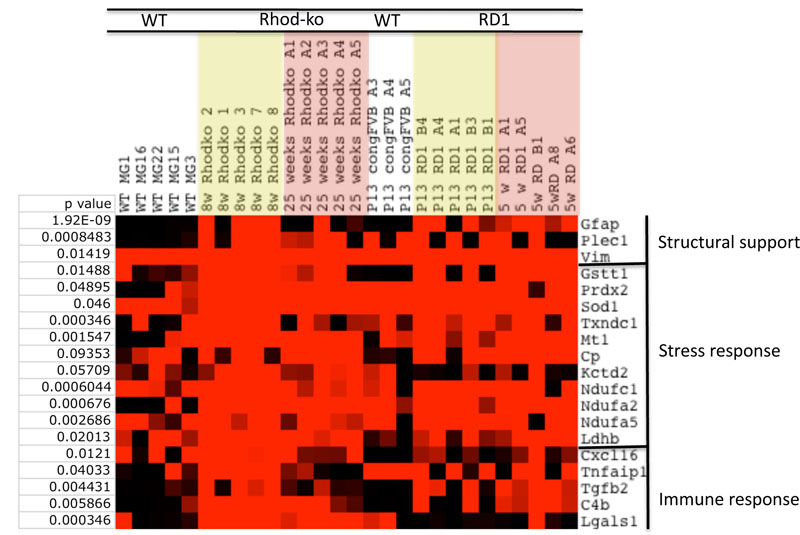
Key features of gliosis. A subset of genes with significant p values (<0.01) compared with *Gfap* expression were distributed into functional groups. Signals from the Affymetrix chip have been scaled such that bright red represents >10,000, and black <1,000, varying shades of red the values in between.

As the retina loses all of its photoreceptors, the outer nuclear layer collapses. However, the inner neural retina remains relatively intact for an extended period, although there are changes in the neuronal processes [50]. Eventually, many cells in the inner layer also die. There is evidence that MGC gliosis may play a role in protecting the retina from the spread of cell death by establishing new boundaries [51]. Indeed, one of the genes with the most prominent association with *Gfap* is plectin (Figure 2), a linker molecule among the three main components of the cytoskeleton and between plasma membrane junctions. Other intermediate and microfilament related genes, such as vimentin and stathmin 1 (a regulatory protein of microtubule dynamics), are highly expressed in MGCs from WT and mutant retinas (Figure 2). High expression levels of intermediate filament proteins and associated proteins might suggest that a stabilization of MGC processes takes place. Interestingly, the intermediate filament nestin, expressed by neural progenitor cells [52], can also be upregulated in a subset of gliotic MGCs (see the full data set in Appendix 1). Integrins, receptors that mediate cell attachment as well as mobility, are also strongly associated with *Gfap* expression (Appendix 2).

Stabilization of MGC processes could additionally be important for the formation of new boundaries between the apical processes of the MGCs and the retinal pigment epithelium (RPE). These boundaries might protect the retina from further damage, such as intrusive pathogenic factors produced by a possibly ruptured RPE. The newly formed boundaries can be considered light scarring between the retina and the RPE [53]. However, no glial scar in the traditional understanding of a central nervous system (CNS) scar is formed. No molecules known to be expressed in reactive astrocytes and microglia have been found in the MGC profiles [54-58]. In this data set, expression of the inhibitory extracellular matrix molecule, CD44 antigen (*Cd44*), was increased in MGCs during cone degeneration in both models (Appendix 1). Chondroitin sulfate proteoglycan, *Cspg5*, was upregulated in a subset of MGCs at early time points and in most cells at later time points. However, no other *Cspg*s are expressed in MGCs. We also found increased hyaluronan expression in the degenerating retina stained with Alcian blue (Alcian Blue 8GX; Sigma–Aldrich, St. Louis MO; Roesch and Cepko, data not shown). All of these data might point to a targeted response within MGCs directed at the extracellular matrix and the structure of MGCs themselves. Indeed, altered expression of adherens junction proteins in the Rhod-ko mouse has been previously reported [59]. Additionally, the outer limiting membrane (OLM) has been shown to be a barrier for PR engraftment in the rat [60,61]. The OLM contains intracellular junctions between PR and MGCs that are defined by expression of *Cd44* [62] and intercellular adhesion molecule 1 (*Icam-1*) [63]. In addition, disruption of the OLM by deletion of the Crumbs homolog 1 (*Crb1*) and zona occludins (*Zo-1*) helps with integrating transplanted PR [60,61].

### Detoxification

During photoreceptor cell loss, MGCs are exposed to a changing environment. Changes likely include an imbalance in ions, oxygen concentration, and reactive oxygen species and inflammation, all of which could potentially be toxic. Genes found to be strongly associated with *Gfap* might thus be “detoxifying” genes. We observed upregulation of the antioxidant gene, glutathione-s-transferase (*Gst1*; Figure 2), that can protect neurons through a reduction in reactive oxygen. Interestingly, MGCs from older mice have been shown to contain a reduced amount of glutathione compared to younger ones [64], which could potentially be a modifier for disease progression in the different models. Transcripts for peroxiredoxins (*Prdx2*) were also found to be upregulated, which could lead to a reduction in H_2_O_2_ and other peroxides in the retinal tissue. Other molecules involved in stress protection with increased expression levels and strong association with *Gfap* were the antioxidant metallothionein 1 (*Mt1*), which binds toxic metals and acts as free radical scavenger, superoxide dismutase 1 (*Sod1*), and genes involved in iron homeostasis, e.g., ceruloplasmin, a copper binding ferroxidase. Nitric oxide synthase was upregulated as well, especially at the early time points in Rhod-ko and rd1 MGCs, even though the synthase did not associate well with *Gfap* expression (Appendix 1). Nitric oxide function at low concentration should be beneficial and protect neurons from glutamate toxicity. Increased nitric oxide synthase expression has been previously shown in response to ischemia and diabetic retinopathy [65,66]. Glutamine synthetase (*Glul*), the only retinal enzyme that can perform ammonia detoxification, was highly expressed in WT MGCs as well as glial cells from the degenerating retinas [67], and therefore did not associate well with *Gfap*. Removal of large molecules from extracellular space by multidrug resistant transporters in the glial membrane has been suggested previously [68]; however, none of these genes were present in the data set. The aforementioned changes in glutathione metabolism, peroxide detoxification, and iron homeostasis could all potentially lead to neuronal protection.

### Growth factors

Neurotrophic factors, growth factors, and cytokines (see below) have previously been suggested to positively affect the survival (and synaptic function) of neurons. MGCs produce the neurotrophins, nerve growth factor (NGF), brain-derived neurotrophic factor (BDNF), ciliary neurotrophic factor (CNTF), basic fibroblast growth factor (bFGF), insulin-like growth factor 1 (IGF-1), glial cell-derived neurotrophic factor (GDNF), leukemia inhibitory factor (LIF), and pigment epithelium-derived factor 318 (PEDF318) [69]. Many of these factors lead to increased glutamate buffering [70,71] and might enhance photoreceptor survival. In the data set presented here, nerve growth factor-β (*Ngfbeta*) expression was high in a subset of WT cells but low in the remaining cells. *Cntf* was highly expressed in early stages of rd1 and Rhod-ko. However, *Cntf* expression was not well correlated with *Gfap*. Other neurotrophic factors, such as *Bdnf*, *bFgf*, *Igf1*, *Gdnf*, *Lif*, and *Pedf*, were not expressed. Further, dickkopf homolog 3 (*Dkk3*) was highly expressed in WT and all Rhod-ko samples (Appendix 1). Interestingly, *Dkk3* expression was low in congenic FVB (WT) at P13, but high in FVB rd1 MGCs at P13. The suggested function of DKK3 is to reduce caspase activity through wingless (Wnt) signaling and to protect against cell death [72,73]. Indeed, it has been previously shown that the Wnt pathway can be activated in MGCs after photoreceptor death [73].

### Homeostasis and energy metabolism

MGCs have been shown to support neurons in a WT situation through their roles in maintaining extracellular homeostasis, including transporting and/or buffering water, potassium ion (K+), glutamate, and other ions [74]. MGCs are proposed to transport waste products, and exchange molecules with retinal blood vessels, vitreous body, subretinal space, and the RPE [4]. Francke et al. [75] showed that depolarized membrane potentials and reduced K^+^ inward conductance indeed do occur in reactive rabbit MGCs, which in turn also suggests an impairment in the clearance of excessive K^+^ ions and neurotransmitter recycling. Interestingly, depolarization of the resting potential seems to be a requirement for astrocyte proliferation [76]. One gene associated with *Gfap* that falls in this category is potassium channel tetramerisation domain containing 2 (*Kctd2*), found to be mainly upregulated in the Rhod-ko MGCs (Figure 2). However, we did not observe any other changes in the expression levels of genes concerned with homeostatic activities.

Another aspect of cellular stress response on the part of glia is the provision of energy to neurons. In the degenerating retina, Punzo et al. [26] have shown that cone photoreceptors appear to be starving. In the healthy retina, it has been proposed that MGCs nourish neurons with lactate and pyruvate that can be used by neurons to make ATP [77], and similar models have been made for other glia elsewhere in the CNS [78]. MGCs can store energy in the form of glycogen and can use glycogenolysis to break it down, depending upon neuronal activity [74]. Indeed, the glia-specific protein, glycogen phosphorylase, is upregulated in a subset of MGCs from degenerating retinas (Appendix 1). Interestingly, MGCs are not dependent on the presence of oxygen and can undergo anaerobic glycolysis [77]. However, when glucose is absent, MGCs can use lactate, pyruvate, glutamate, or glutamine to make energy [79]. Many transcripts involved in energy metabolism, such as glucose phosphate isomerase 1, lactate dehydrogenase A, and pyruvate kinase, were slightly upregulated or were steady across most Rhod-ko- and rd1-derived MGCs (Appendix 1).

### Immune response and inflammation

Efficient elimination of apoptotic cells during degeneration is assumed to be crucial for regulating the immune response and preventing a potentially devastating inflammatory reaction in all tissues. The retina is traditionally looked at as an immune-privileged site [80]. However, the retina carries out an innate and adaptive immune response [81-83]. The innate immune response relies largely on phagocytic cells–expressing receptors that recognize molecular patterns on apoptotic cells to distinguish them from living cells. After binding to apoptotic cells, anti-inflammatory signals and other immunomodulatory cytokines are released by the cells of the immune system, but perhaps, as discussed below, also by MGCs. MGCs are thought to be important mediators of short-range interactions among retinal cells [4], and therefore, MGCs might act as modulators of the immune and/or inflammatory response and might spatially and temporally restrict it. MGCs could be the cell type that recognizes the death of photoreceptors and might communicate this to other cell types in the retina. MGCs might also send the signals that recruit microglia. Alternatively, or additionally, microglia that migrate into the retina could release molecules that trigger gliosis in MGC.

Indeed, upregulation of *Gfap* in MGCs is associated with recruitment of microglia into the retinal tissue. Usually, astrocytes and microglia in the healthy retina are confined to the innermost retinal layers (nerve fiber layer, ganglion cell layer, inner plexiform layer). Monocyte chemoattractant protein 1 (MCP-1, CCL-2) expression was shown in MGCs after retinal detachment [84,85]. MGCs also secrete the proinflammatory cytokine tumor necrosis factor α (TNF-α) [86-89]. MCP-1 and TNF promote infiltration by immune cells. Interestingly, there was upregulation of TNF-α induced protein 1 (*Tnfaip1*) in MGCs from the RP retinas, suggesting that MGCs are responsive to it as well (Figure 2). The antioxidant, heme oxygenase, can decrease recruitment of immune cells into retinal tissue, and its transcript was observed to be upregulated in MGCs during degeneration [90] (Appendix 1). It is possibly also regulated by *Icam-1*, which shows increased expression levels in a subset of MGCs from RP retinas [91]. Other chemotactic factors for macrophages and monocytes upregulated in the single MGCs are monocyte to macrophage dissociation associated 2 (*Mdm2*) and macrophage migration inhibitory factor (*Mif1*). Also upregulated was the transcript for CCAAT/enhancer binding protein delta (*Cebpd*), a protein that regulates macrophage activation genes. See the full data set in Appendix 1 for detailed expression on all of these.

In the data set presented here, there are indications of an immune-related response. Some MGCs showed upregulation of cytokine and cytokine receptor genes, as well as complement components. Some components of the chemokine system (*Xcr1* and *Cxcl16*) also can be detected in WT MGCs [27]. *Cxcl16* showed significant association with *Gfap* (Figure 2). Several other small inducible cytokines and their receptors (*Ccrl1*, chemokine C-C motif receptor like 1 and *Ik*, IK cytokine) were upregulated, too, however with less significance than *Gfap*. Also strongly associated with *Gfap* was complement component 4B. MGCs express the receptor for complement C3a, and complement regulatory proteins CD55 and CD59 [92]. However, they do not seem to be upregulated in our data set. Previous data also showed that astrocytes provide information (intercellular exchange of signaling molecules) to MGCs via gap junctions [93,94]. However, tracer molecules are not passed from MGCs to astrocytes [95,96]. Certain steps (e.g., initiation) might depend upon MGCs interacting with immune cells such as microglia [9] and might modify the amount of neurotrophic factors produced by MGCs, which in turn can modify neuronal survival (see the stress response section below).

MGCs might even be more directly involved in the prompt clearing of photoreceptor debris. MGCs could phagocytose PR cell debris. Their phagocytic ability has been previously shown in vitro (latex beads) and in vivo (melanin) after retinal detachment [97-99]. During Drosophila development, glial cells engulf degenerating axons through recognition mediated by Drpr/Ced-1 and Ced-6/CED-6 [100]. Researchers have proposed that apoptotic cells and degenerating axons of mature neurons are removed by a similar mechanism [100,101]. Interestingly, MGCs from degenerating retinas in both models expressed *Lgals3* (galectin-3, Appendix 1), a gene known to be expressed in phagocytic cells and recently shown to be expressed during glaucoma by astrocytes in the optic nerve head [102]. However, none of the prominent genes in this pathway were found closely coexpressed with *Gfap*.

### Cell cycle reentry and proliferation

**Figure 3:**
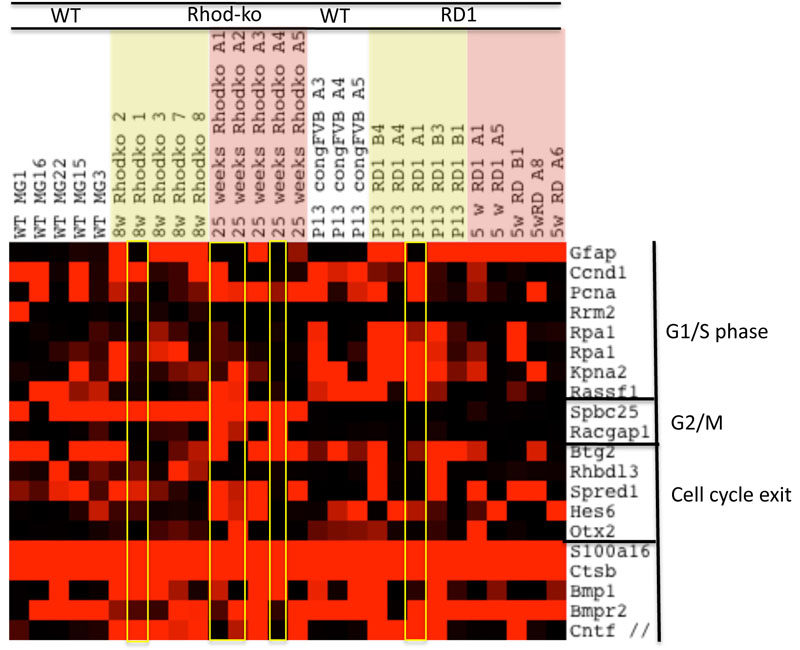
Gliosis-cell cycle reentry and proliferation. A Java TreeView-generated heatmap illustrating the expression of genes involved in control of the cell cycle and proliferation. Signals from the Affymetrix chip have been scaled such that bright red represents >10,000, and black <1,000, varying shades of red the values in between.

Cells undergoing reactive gliosis raise the concern of tumor formation. However, no glial tumors have been observed in the retina of humans or animals. This suggests a tightly regulated process of proliferation that safeguards the retina. In chicks, cells with increased *Gfap* expression in response to damage or exogenous growth factors did not reenter the cell cycle [48]. In the data set presented here, a subset of MGCs, one Rhod-ko cell picked during the rod death phase, four cells picked during the cone death phase, and one rd1 cell during the rod death phase, showed low to no *Gfap* expression (Figure 3). Higher expression levels for cell cycle genes were observed in two of the Rhod-ko *Gfap* negative cells (A1 and A2) and in one P13 rd1 *Gfap* negative cell (A1). For the latter, this might just be a general feature of a cell in a still immature retinal environment. Across the rest of the cells, cell cycle gene expression remained mostly low and mosaic (Figure 3). Transcripts for inhibitors of cell cycle progression, differentiation, and microtubule assembly (S100 calcium binding protein A1, A16 and their interacting protein cathepsin B) were upregulated. Expression levels of genes associated with proliferation were low, e.g., cyclin D1 and proliferating cell nuclear antigen (*Pcna*) (Figure 3). In the CNS, glial cells express antiproliferative molecules, e.g., BMP and CNTF, even in a WT situation [103,104]. In the MGC data presented here, *Bmp1* was upregulated in the Rhod-ko, and *Bmpr2* was highly expressed across mutants. *Cntf* was upregulated at early time points in Rhod-ko and rd1, and in some cells in rd1 at later time points. The transcript for *Cntfr* was upregulated in most of the mutant cells, and was low in WT MGCs. Other antiproliferative genes such as *Spbc25*, *Pea15*, and *Btg2* were found to be highly expressed in the (majority of) WT cells and stayed highly expressed in Rhod-ko at week 6 and week 25 (Figure 3). Surprisingly however, *Spbc25* was not expressed in any of the RD1 MGCs (Figure 3). The complete absence of this gene in rd1 might be a reflection of genetic differences in the strain background for rd1.

These data, as well as previously published results [48,105], suggest that an induction of proliferation is not the case during photoreceptor cell loss in RP. A more severe or acute insult might be needed to trigger this activity [106]. Massive proliferation has indeed been shown to lead to proliferative gliosis, depolarization, and uncontrolled proliferation, and to result in glial scar formation and further death of retinal tissue after vitreal injection of hemoglobin, during proliferative vitreoretinopathy, proliferative diabetic retinopathy, and retinal detachment [107-109]. Additionally, proliferation and dedifferentiation of MGCs can also be triggered in a WT situation after stimulation with Wnt3a and drugs such as retinoic acid and valproic acid [47].

### Progenitor cells and neural repair

**Figure 4:**
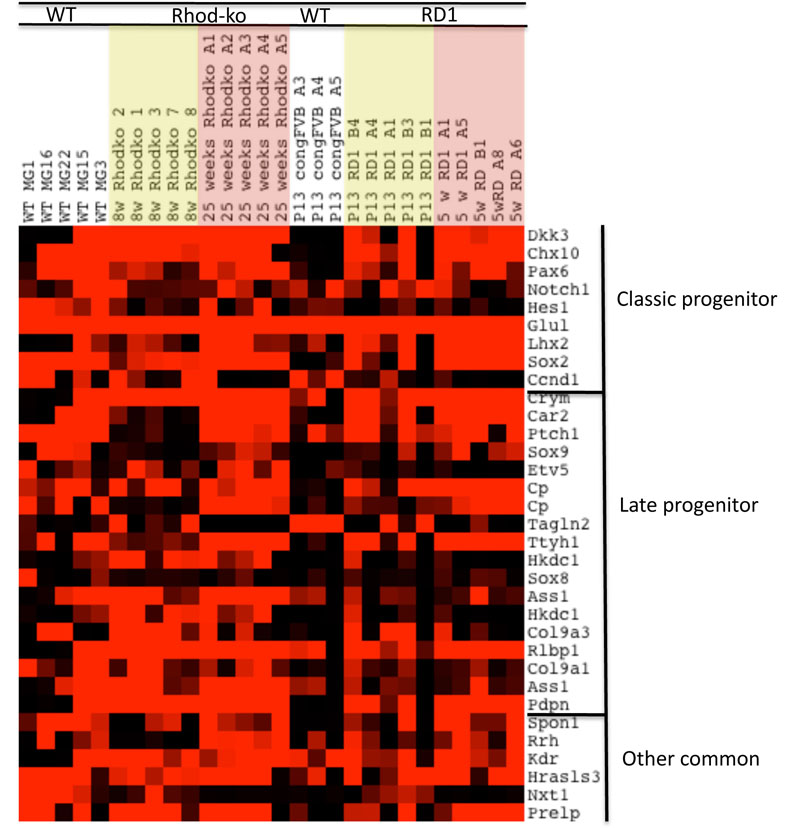
Gliosis–progenitor cell potential. A heatmap created in Java TreeView displaying the expression of selected progenitor cell genes across the MGC samples. Signals from the Affymetrix chip have been scaled such that bright red represents >10,000, and black <1,000, varying shades of red the values in between.

MGCs have been suggested to have neurogenic competence and to be a potential source of neural regeneration. In zebrafish, MGCs indeed are able to regenerate all retinal cells and architecture following several types of insult [34,36,38,110]. Mature MGCs have previously been shown to have a similar expression profile to late retinal progenitor cells [27,111]. A late progenitor cell likely “morphs” into an MGC without undergoing an irreversible cell fate change, whereby the progenitor cell activates the genes necessary for MGC function but does not lose its progenitor traits [112]. Indeed, many progenitor cell genes are expressed heterogeneously across WT MGCs. Classic progenitor and late progenitor genes are more highly expressed and expressed in more cells, at the later disease stage of cone death (Figure 4). Interestingly, most of the transcripts for classic progenitor genes are expressed at low levels in WT MGCs (congenic FVB) at P13, with the exception of *Glul* and transgelin2 (*Tagln2*), which are highly expressed in all three cells picked. This result might reflect that the possibility that MGCs are maturing at P13, where they require a very specific set of genes to fully carry out their maturation.

Dedifferentiation of MGCs to progenitor cells after photoreceptor cell loss might potentially involve functional uncoupling from neurons and a disruption of neuron-glia interaction and homeostasis. Essential MGC functions, such as neurotransmitter recycling (*Glul*), carbon dioxide (carbonic anhydrase) and potassium siphoning, visual pigment cycling (*Cralbp*), glycolysis, and water regulation could be affected [109,113,114]. However, the genes involved in these functions are unchanged at the transcriptional level. Transcripts of glial markers such as carbonic anhydrase 2, retinaldehyde-binding protein (*Cralbp*), aquaporin 4, and glutamine synthase (*Glul*) stay highly expressed across most MGCs (Appendix 6). However, dedifferentiation could occur through mislocalization of K^+^-channels and reduced conductance [106] and result in the loss of function of neuron-glia interaction (which requires a hyperpolarized membrane). Such changes on a protein expression or localization level would obviously not be identified on the RNA level as studied here.

From a therapeutic point of view, MGCs remain available for “modifications” after photoreceptor cell loss and could be used for therapeutic strategies for retinal repair. MGCs could be stimulated to reenter the cell cycle, express photoreceptor specific genes, and potentially even undergo neurogenesis to replace the lost photoreceptor cells. However, MGCs’ in vivo potential to do so in mammals is ambiguous. At this point, it is also unclear how well newly produced photoreceptors integrate and function. However, several recent studies of engrafted photoreceptor cells suggest that the retina is indeed able to respond to light following engraftment [115,116].

In consideration of the possibility that MGCs might be able to regenerate photoreceptor cells, we compared the RP and WT MGC microarray data to profiling data from regenerating zebrafish tissues, including the retina [106], heart [117], and fin [118]. Data from regenerating tissues might help identify molecular triggers needed for MGCs’ acquisition of stem cell properties. Interestingly, many shared upregulated genes across three studies of regeneration in zebrafish fall into the categories of cell signaling, immunoregulation, regulation of transcription, and stress response. These three categories are discussed above in the context of RP, and a more detailed comparison revealed the following similarities.

**Figure 5:**
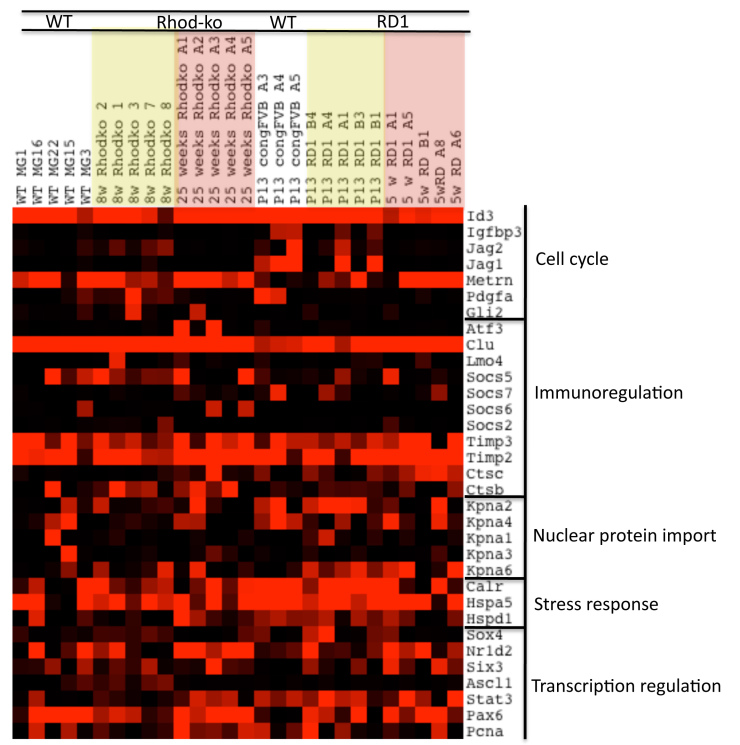
Gliosis–regenerative potential. A heatmap created in Java TreeView shows the expression of a selection of genes common to regeneration across MGCs. Signals from the Affymetrix chip have been scaled such that bright red represents >10,000, and black <1,000, varying shades of red the values in between.

The proliferation marker *Pcna* (proliferating cell nuclear antigen) was highly expressed in most MGCs at the late time point in the Rhod-ko as well as in a subset of glia at the early time points in both models, but surprisingly, also in a subset (two of five) of WT MGCs. Kassen et al. [119] noticed that *Stat3* expression increased after light-induced PR death in zebrafish and that a subset of *Stat3* expressing cells also express *Pcna*. The same observation was made for the MGC data set (Figure 5). The immunoregulatory gene, *Atf3,* was expressed in two out of five cells at week 25 in Rhod-ko. Interestingly, those cells also expressed *Pcna*. Other immunoregulatory genes such as *Socs* members and the tissue inhibitor of metalloproteinase (*Timp*) were heterogeneously expressed. Karyopherin alpha 2 (*Kpna2*), a gene associated with protein import into the nucleus, which is expressed in retinal progenitor cells during the S phase [49], showed high expression values in the rd1 at P13. *Nr1d2*, a gene involved in regulating transcription, was highly expressed at later time points in both models and heterogeneously at early time points, but not in P13 WT. Further work on the function of these genes, potentially in the entry to cell cycle, will be needed to interpret these intriguing changes.

*Ascl1a* (proneural bHLH) and *Six3b* (early eye development) are transcription factors expressed in zebrafish retinal progenitors and upregulated in the data set of Qin et al. [117]. *Ascl1* was expressed in two WT cells and three of five Rhod-ko cells during rod death. The levels were low (fewer than 2,000), but since values within the other cells fluctuated near 0, this may represent a significant upregulation. *Six3* also was expressed heterogeneously across MGCs from degenerating retina. As a side note, the transcription factor *Pax6*, a retinal progenitor gene marker, was also expressed in a subset of MGCs [27,120], and interestingly, a subset of those *Pax6* positive cells also were *Pcna-*positive. In keeping with this correlation, a subset of PCNA-positive cells also were *Pax6* positive (three out of nine cells). *Sox4a*, a gene usually expressed in committed neural progenitors, was upregulated in one week 25 Rhod-ko MGC, and most of the rd1 P13 cells. The negative regulator of bHLH genes, *Id3*, stayed high in all of the MGCs. Also highly expressed in most of the cells from degenerating retina as well as in a subset of WT cells at the adult stage and all of the WT cells at P13 were the stress response genes, *Hspd1* (mitochondrial chaperone Hsp60), *Hspa5* (*Hsp70*), and calreticulin. These similarities between the murine MGC data set and the zebrafish data imply that MGCs indeed share some features with cells that are able to regenerate their tissues. It would be interesting to know if only a sub-population of MGCs was maintained to potentially produce neurons later or if all MGCs could be induced to do so.

### Inverse correlation with *Gfap*

**Figure 6:**
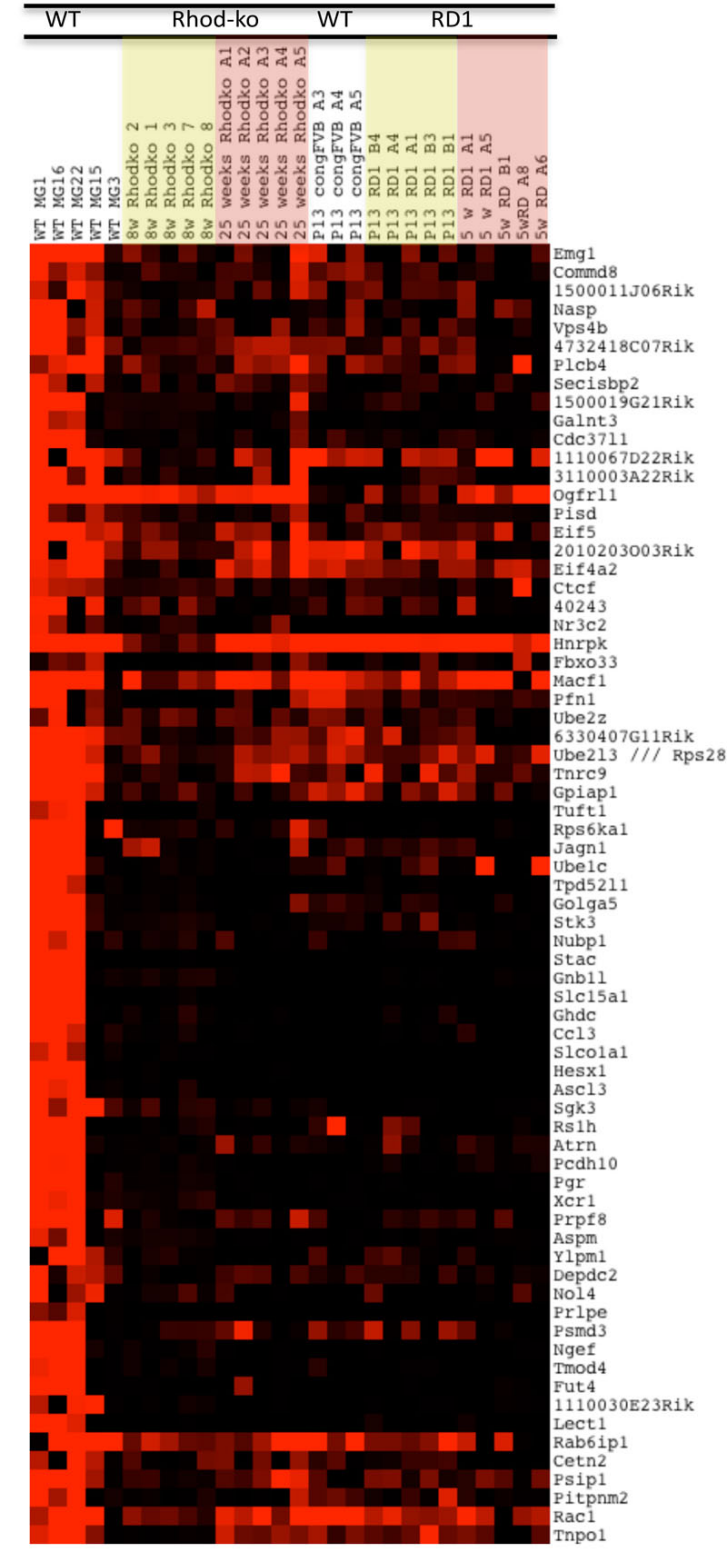
Gliosis–transcriptional profiles of genes associated with *Emg1*. All the top hits for genes coregulated with *Emg1* are shown in a heatmap. Signals from the Affymetrix chip have been scaled such that bright red represents >10,000, and black <1,000, varying shades of red the values in between.

**Figure 7:**
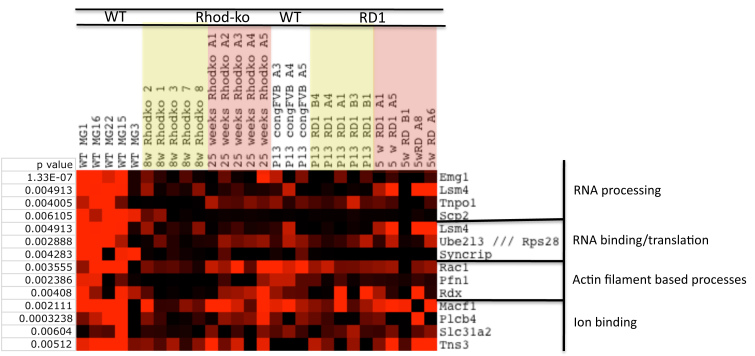
Gliosis–gene ontology grouping of genes associated with *Emg1*. A subset of these differentially expressed genes was grouped according to their function in a biologic processes.

Many genes appeared to be inversely correlated with *Gfap*. The gene *Emg1* (ribosome synthesis factor/ methyltransferase) was one such gene. We found 1,375 genes coregulated with *Emg1*, out of which 573 had p values of <0.01 (Figure 6 and Appendix 7). Function–based analysis performed with the DAVID functional annotation tool revealed enrichment for the following categories (Figure 7): RNA binding and translation (*Lsm4*, *Eif4g2*, *Eif5a*, *Rps28*, *Syncrip*), regulation of actin filament based processes (*Rac1*, *Pfn1*, and *Rdx*), RNA processing/acetylation (*Emg1*, *Lsm4*, *Tnpo1*, *Ube2l3*, *Pfn1*, *Rdx*, *Rsp28*, *Scp2*, *Syncrip*), catabolic process/proteolysis (*Ube2l3*, *Ube2z*), and ion binding (*Macf1*, *Plcb4*, *Slc31a2*, *Tns3*). As discussed earlier, glial cells are crucial in modifying the activation thresholds and plasticity of neurons as well as their communication, which might be particularly important during photoreceptor cell death. A highly controlled network for RNA processing might allow them to do so. Two genes involved in regulatory mRNA pathways, *Syncrip* (mRNA transport and stabilization) and *Lsm4* (mRNA decapping and degradation), have been previously shown to be expressed in glial cells [121]. Zebrafish regeneration studies [121] showed a predominant number of downregulated genes (60%) in categories such as cell communication, signal transduction, and transcription regulation during cell death and regeneration. As one would predict, RNA processing has been shown to be involved in controlling differentiation from neural stem cells [120], and RNA splicing has been previously shown to be an important process for adult neurogenesis [122]. Additionally, downregulation of *Rac1* in the CNS allows for regeneration in the sciatic nerve [123]. The observed downregulation of these genes involved in mRNA processing and transport in MGCs may therefore reflect a reduced differentiation potential [124], which is consistent with the absence of proliferation and dedifferentiation during photoreceptor cell death in RP. This will be a critical issue to address to be able to achieve progress in modifying MGCs as a therapeutic approach for retina degenerations.

### Conclusions

The data reported here provide a starting point for analyses of MGC function during retinal degeneration. Many hypotheses are possible given the number of functions likely performed by MGCs in normal and degenerating retinas. Although the heterogeneity inherent in WT MGCs, as well as the potential heterogeneity in response to injury, makes a statistical analysis and validation difficult to perform, functional tests for some pathways are suggested by the data discussed above.

## Appendix 1. Full Affymetrix data set.

Signal levels of all 28 MGC arrays are represented in an Excel table. To access the data, click or select the words “Appendix 1.” This will initiate the download of a compressed (pdf) archive that contains the file. Signals from microarrays were processed by using Affymetrix Suite Software (MAS 5.0). The resulting data was exported as tab delimited text file. WT=wildtype, Rhod-ko=Rhodopsin knockout model, rd1=rd1 model, and cong FVB=congenic FVB. Time points chosen are > 21 days for WT, and 8 weeks and 25 weeks for the peaks of the two cell death waves in the Rhod-ko model. The corresponding time points in the rd1 are postnatal day (P) 13 and 5 w. MGCs from congenic FVB at P13 were also profiled as WT samples to closer age match the early cell death wave in the rd1 model.

## Appendix 2. Median expression distribution.

To access the data, click or select the words “Appendix 2.” This will initiate the download of a compressed (pdf) archive that contains the file. Multiple probe sets corresponding to a single gene were combined by selecting whatever probe set gave the highest value in a particular cell, and their median expression was calculated. From the distribution of median expression levels over all cells on the x-axis (logarithmic) an initial peak of low expressed genes (probably off) on the left can be observed followed by a tail on the right side (probably on). Red lines represent chosen threshold levels.

## Appendix 3. P value distribution for genes coregulated with the gliosis marker *Gfap.*

To access the data, click or select the words “Appendix 3.” This will initiate the download of a compressed (pdf) archive that contains the file. *Gfap* is represented with a green line. Other genes are shown for comparison. x-axis=p value threshold, y-axis=fraction of genes.

## Appendix 4. Genes with similar expression patterns to *Gfap*.

To access the data, click or select the words “Appendix 4.” This will initiate the download of a compressed (pdf) archive that contains the file. Multiple Affymetrix numbers for the same probeset (the same gene according to Affymetrix annotation) were collapsed using the maximum value for each array. Significance cut-off for P value was chosen as <0.01, corresponding to 281 gene hits. The first three columns contain the information on the genes. The remaining column contains the p-values for co-regulation between the gene in that row and the gene in the column title.

## Appendix 5. P values of genes associated with *Ccnd3*.

To access the data, click or select the words “Appendix 5.” This will initiate the download of a compressed (pdf) archive that contains the file. The cut off was set at <0.01. Gene information (Affymetrix ID and gene name) is shown in the first three columns. The remaining column contains the P values for co-regulation between the gene in that row and *Ccnd3*.

## Appendix 6. P values of genes associated with *Chx10*.

To access the data, click or select the words “Appendix 6.” This will initiate the download of a compressed (pdf) archive that contains the file. The cut off was set at <0.01. Gene information (Affymetrix ID and gene name) is shown in the first three columns. The remaining column contains the P values for co-regulation between the gene in that row and *Chx10.*

## Appendix 7. Genes with inverse expression patterns to *Gfap*.

To access the data, click or select the words “Appendix 7.” This will initiate the download of a compressed (pdf) archive that contains the file. *Emg1* was chosen as co-regulation marker. The significance cut off was set at 0.01. Gene information (Affymetrix ID and gene name) is shown in the first three columns. The remaining column contains the P values for co-regulation between the gene in that row and *Emg1*.
